# Protective effect of aqueous and methanolic extracts of corn silk on nicotine-induced reproductive system disorders in male mice

**DOI:** 10.5935/1518-0557.20230034

**Published:** 2023

**Authors:** Mohammad Sa’adatzadeh, Ali Akbar Oroojan, Mohammad Amin Behmanesh, Maysam Mard-Soltani

**Affiliations:** 1 Faculty of Medicine, Dezful University of Medical Sciences, Dezful, Iran; 2 Department of Physiology, Faculty of Medicine, Dezful University of Medical Sciences, Dezful, Iran; 3 Department of Histology, Faculty of Medicine, Dezful University of Medical Sciences, Dezful, Iran; 4 Department of Clinical Biochemistry, Faculty of Medicine, Dezful University of Medical Sciences, Dezful, Iran

**Keywords:** nicotine, corn silk, reproduction, mouse

## Abstract

**Objective:**

The protective effect of aqueous and methanolic extracts of corn silk on reproductive disorders induced by nicotine was investigated in the present study.

**Methods:**

In this experimental study, 30 male NMRI mice (25-30gr) were divided into 5 groups: controls, sham, nicotine 2.5mg/kg, nicotine+aqueous extract of corn silk 400mg/kg, and nicotine+methanolic extract of corn silk 400mg/kg for 34 days. One day after the last nicotine and extracts administration, the serum samples were collected through cardiac puncture for hormonal measurements, and the testis and tail of the epididymis were isolated for the testis antioxidant, morphology, histopathology assessments, and sperm count.

**Results:**

Luteinizing hormone (LH) and malondialdehyde (MDA) increased in the nicotine group. Testosterone, sperm count, and glutathione (GSH) decreased when compared to the control group. Both aqueous and methanolic extracts of corn silk led to the improvement of mentioned changes; Except for GSH, because only treatment with methanolic extract could lead to its increase (*p*<0.05). Nicotine decreased the thickness of the epithelium of seminiferous tubules and the separation between them, and the administration of corn silk extracts improved that.

**Conclusions:**

Nicotine consumption increased oxidative stress, LH levels, and decreased testosterone and sperm count, which indicate the induction of primary hypogonadism in animals. Moreover, the use of corn silk extracts has recovered the amounts of sex hormones and sperm count to normal conditions by reducing lipid peroxidation.

## INTRODUCTION

Nicotine is an important alkaloid found in cigarettes, which has the ability to produce many harmful effects on the body. Nicotine contained in cigarette tobacco can impair reproductive processes such as spermatogenesis, semen or sperm concentration or volume, and sperm motility. Moreover, this compound is an endocrine disruptor, which increases sperm DNA fragmentation, mutagenesis, and aneuploidy. Finally, these alterations lead to decrease in male fertility (Omolaoye *et al*., 2022). Nicotine easily passes through the membrane of body cells and has the ability to react with some intracellular components such as tubulin protein in the cytoplasm of dividing cells such as germ cells. It has been found that the consumption of nicotine causes disruption in the spermatogenesis process; atrophy and damage to the testicles through induce oxidative stress and increase reactive oxygen radicals (ROS) production in mice (Jalili *et al*., 2021). The balance of the oxidant and antioxidant defense systems is essential for maintaining sperm functionality. If this balance is disturbed, it can cause the generation of ROS and oxidative damage to sperm. Due to the presence of polyunsaturated fatty acids (PUFAs) in the cell membrane of spermatozoa, this cell is very susceptible to oxidative destruction. During pathological conditions, ROS can induce damage in multiple biomolecules of sperm including nucleic acids, proteins, and lipids. Finally, these changes cause loss of membrane integrity, mitochondrial dysfunction, impaired sperm motility as well as DNA damage and apoptosis (Ayad *et al.*, 2022). Also, in humans, the use of nicotine agonists by acting on acetylcholine receptors reduces the production of androgens (Arabi & Anand, 2002). Male infertility has various causes, including genetic mutations, lifestyle, various diseases, and medication consumption. “As shown in International Committee for Monitoring Assisted Reproductive Technology (ICMART) and the World Health Organization (WHO), more than 186 million people worldwide suffer from infertility. Research has shown males are responsible for 20-30% of infertility cases but contribute to 50% of cases overall” (Balawender & Orkisz, 2020). Different types of diseases such as kidney disease, liver failure, hemochromatosis, chronic obstructive pulmonary disease, and cystic fibrosis affect sperm parameters. These factors can affect male fertility through different mechanisms, which include affecting hormonal levels, sexual dysfunction, or testicular dysfunction and spermatogenesis (Fainberg & Kashanian, 2019). Nicotine consumption has a negative effect on spermatogenesis, epididymal sperm count, motility, and fertility potential. Nicotine also disrupts the function of Leydig cells and reduces testosterone production (Ni *et al*., 2020; Salahipour *et al*., 2017).

In recent years, numerous medicinal plants have been studied for the improvement of male infertility. Due to the richness of medicinal plants in antioxidants, attention to their use in male infertility is increasing in recent times (Aldaddou *et al*., 2022). Corn silk is considered a waste product from corn cultivation (Yang *et al*., 2014). This part of the corn plant has been used in hundreds of dietary therapies for over thousands of years. In addition, its safety and non-toxicity have been approved by the United States Food and Drug Administration (FDA). One study investigated the effect of different fractions of ethanolic extract of corn silk on diabetes and diabetic nephropathy, the results of which indicated that the ethyl acetate fraction (ECS) and n-butanol fraction (BCS) have the highest activity. Total antioxidant had the strongest inhibition effect against hydroxyl radicals. ECS and BCS showed significant hypoglycemic effects with significant inhibition of α-amylase and α-glucosidase in enzyme assays. Also, ECS and BCS effectively inhibit the formation of advanced glycation end products (AGEs). Furthermore, investigating their anti-diabetic nephropathy activity showed that ECS and BCS significantly inhibited the production of interleukin-6 (IL-6) in mesenchymal cells stimulated with high glucose. These findings suggest that the antioxidant activities of corn silk can at least partially contribute to the claimed therapeutic benefits in diabetes and its resulting nephropathy. Finally, fractions enriched with phenolic compounds of corn silk ethanolic extract can be considered as a source of natural antioxidants and can be used to prevent and treat diabetes and its complications, including diabetic nephropathy (Wang & Zhao, 2019). Another study, with rats with benign prostatic hyperplasia showed that the use of corn silk ethanolic extract can inhibit cell proliferation by inhibiting the expression of 5-alpha reductase mRNA and reducing the concentration of 5-alpha reductase, dihydrotestosterone and prostate specific antigen (PSA) that leads to the improvement of this disorder (Kim *et al*., 2017). In a study conducted by Kim *et al.* (2017) on large male laboratory mice, they reported that many flavonoids extracted from corn silk have strong antioxidant activities in laboratory conditions, and these compounds have been proposed to increase the level of antioxidant enzymes and inhibit lipid peroxidation in small laboratory mice (Hu & Deng, 2011; Ren *et al*., 2013). Therefore, considering the prevalence of nicotine consumption in human society and its negative effect on the reproductive system through increasing oxidative stress and reducing the antioxidant defense function, and the effect of corn silk on improving this defense system in different parts of the body. In the present study, we decided to investigate the protective effect of aqueous and methanol extracts from corn silk against reproductive disorders induced by nicotine in male mice.

## MATERIALS AND METHODS

In this experimental study, 30 adult male Naval Medical Research Institute (NMRI) mice (6-8 weeks old, 25-30 g) were obtained from the Animal Reproduction and Maintenance Center of Dezful University of Medical Sciences and kept in a 12-hour light-dark cycle, with free access to commercial chow and tap water. This study was approved by the ethics committee of the Dezful University of Medical Sciences with ethics committee grantee No. IR.DUMS.REC.1400.014.

### Plant extraction

Corn silk is a common *Stigma maydis*, purchased from a reputable perfumery in Ahvaz, Khuzestan, Iran. In order to prepare corn silk aqueous extract, 100 g of the powder of this part of the plant was poured into 1 liter of distilled water and mixed in a shaker for 48h. After passing through the filter, it was centrifuged at 3500 rpm for 20 min (Saheed *et al*., 2015). For the sake of methanolic extract preparation, 250 g of corn silk powder was mixed in 1 liter (80-20%) of distilled water and methanol for 72 h, and similarly to the method of preparing aqueous extract, after passing through a filter it was centrifuged at 3500 rpm for 20 min (Wans *et al*., 2021). Finally, after drying the supernatant solution of both extracts in a 37°C incubator, the resulting powder was kept at 4°C until use.

### Preparation of the animals

The animals were divided into five groups as follows (the number of mice in each group was 6) (Aldaddou *et al*., 2022):

1. Control.
2. Sham (received normal saline through intraperitoneal injection and gavage).
3. Nicotine (received nicotine (2.5 mg/kg) by intraperitoneal injection) (Salahshoor *et al*., 2016).
4. Nicotine (2.5 mg/kg) + aqueous extract of corn silk 400 mg/kg orally (Saheed *et al*., 2015).
5. Nicotine (2.5 mg/kg) + methanolic extract of corn silk 400 mg/kg orally (Wans *et al*., 2021).
6. Considering that a spermatogenic cycle in mice is 34 days, the duration of using nicotine and extracts was 34 days simultaneously (Ernst *et al*., 2019).

### Hormonal and antioxidant measurements

One day after the last dose of nicotine and extract administration, the animals were subjected to deep anesthesia with the ketamine-xylazine combination (70-10 mg/kg), then blood samples were collected by cardiac puncture and serum samples were separated from that. The concentrations of luteinizing hormone (LH), follicle stimulating hormone (FSH) and testosterone hormones were analyzed using the enzyme-linked immunosorbent assay (ELISA) method in commercial kits. On the other hand, the right testis of mice was homogenized using phosphate buffer saline, and after centrifugation at 5000 rpm for 10 minutes, the supernatant sample was used to measure malondialdehyde (MDA) and glutathione (GSH) levels.

### Measurement of testicular morphology

The left testicle of the animal was separated and its weight, small and large diameters were measured. Also, the testicle volume was calculated using the formula V= (d^2^ ×π/4) L × K. In this formula, V is the volume, d is testis width, L is testis length, π=3.14, and k=0.9 (constant coefficient) (Oroojan *et al.*, 2021).

### Sperm count assessment

After macroscopic observations, in order to count the sperms in all groups, the cauda epididymis was separated and placed in 6 mL of distilled normal saline serum and minced into small pieces. After stirring the mixture, 10 µL of it was placed in a hemocytometer counting chamber. Then, sperm count was assessed by using the white blood cells counting method (Oroojan *et al.*, 2021).

### Testicular histology

The left testis of the animals was fixed in 10% formalin after separation. After histological processing, 5-7-micron paraffin sections were prepared, the sections were stained with hematoxylin and eosin and histological changes were examined under a light microscope. From each animal, at least 6 slides were considered for each testis (Oroojan *et al.*, 2021).

### Statistical analysis

The data were statistically analyzed using the SPSS software (version 22; SPSS Inc., Chicago, Ill) with one-way analysis of Variance (ANOVA) followed by Post hoc least significant differences (LSD) test. The results were presented as the mean ± standard errors (SEM). *p*<0.05 was considered statistically significant.

## RESULTS

### Effect of aqueous and methanol extracts of corn silk on body weight and testis morphology

**Figure 1:**
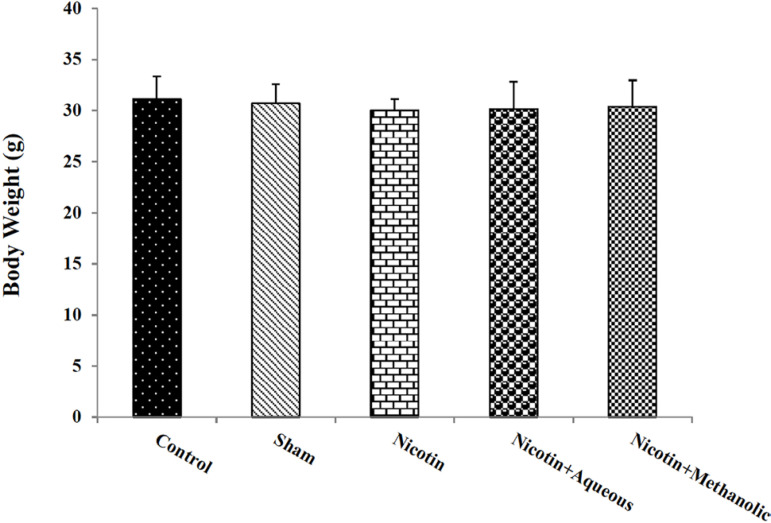
Effect of aqueous and methanol extracts of corn silk on body weight. The results are Mean±SEM for 6 mice in each group.

The present results showed that there was no significant difference in body weight in the mice from different groups (Figure 1). Also, the results of the weight and morphology of the testes indicated that there was no significant difference in the investigated variables between the studied groups. Although the testis volume showed a tendency to increase in the treated groups with aqueous and methanol extracts of corn silk, this increase was not significant (Table 1).

**Table 1:** The effects of aqueous and methanolic extracts of corn silk on the weight and morphology of the testis.

Variables	Testis weight (g)	Testis length (mm)	Testis width (mm)	Testis volume (mm^3^)

Groups				
Control	0.096±0.003	7.80±0.37	5.20±0.20	148.64±9.22
Sham	0.085±0.007	7.60±0.24	5.20±0.37	148.65±10.98
Nicotine	0.101±0.006	7.57±0.20	5.14±0.14	142.61±10.67
Nicotine + Aqueous extract	0.100±0.005	7.50±0.28	5.50±0.28	163.55±13.04
Nicotine + Methanolic extract	0.099±0.004	7.55±0.29	5.33±0.23	157.70±12.94

The results are Mean±SEM for 6 mice in each group.

### Effect of aqueous and methanol extracts of corn silk on serum LH, FSH, and testosterone levels

**Figure 2:**
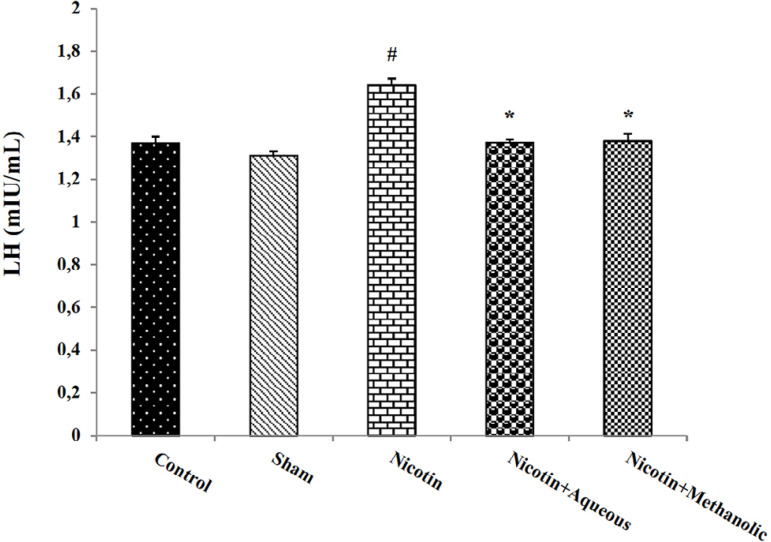
The effect of aqueous and methanol extracts of corn silk on serum LH hormone levels. The results are Mean±SEM for 6 mice in each group. #: *p*<0.05 compared to the control group; *: *p*<0.05 compared to nicotine group.

**Figure 3:**
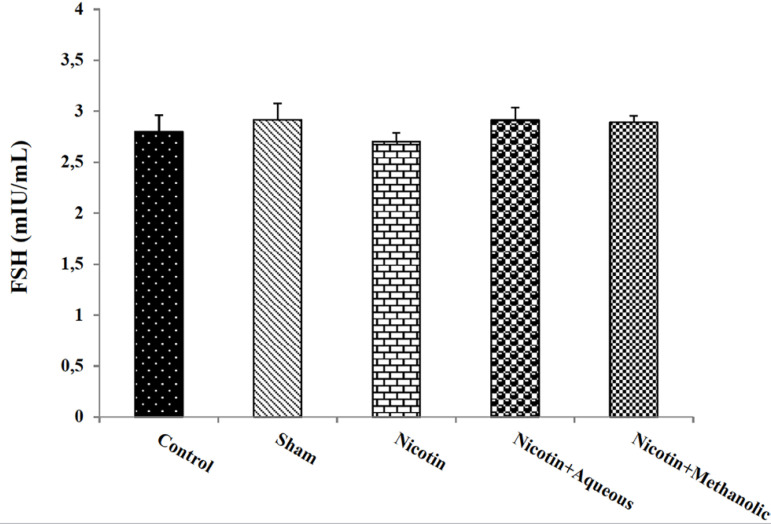
The effects of aqueous and methanol extracts of corn silk on the amount of serum FSH hormone. The results are Mean±SEM for 6 mice in each group.

**Figure 4:**
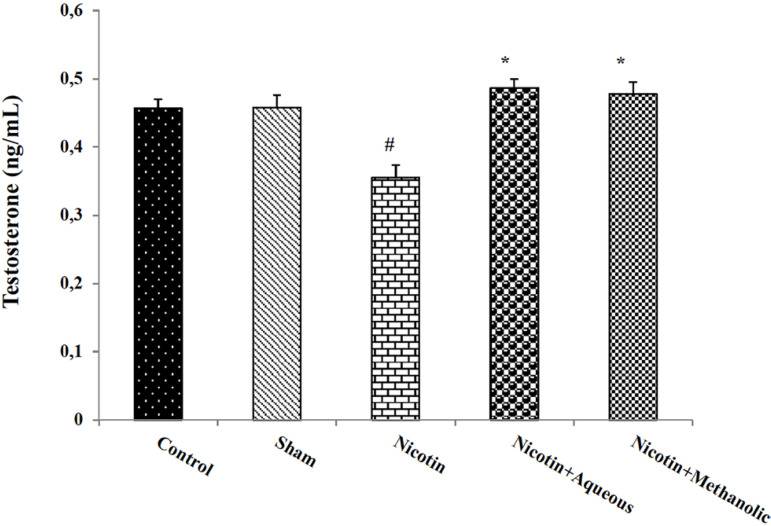
The effects of aqueous and methanol extracts of corn silk on serum testosterone levels. The results are Mean±SEM for 6 mice in each group. #: *p*<0.05 compared to the control group; *: *p*<0.05 compared to the nicotine group.

The amount of LH hormone in the serum of the nicotine-injected mice increased significantly compared to the control group (*p*<0.05). This hormone level showed a significant decrease in the nicotine when treated with aqueous and methanol extracts of corn silk in comparison with the nicotine group (*p*<0.05; Figure 2). There was no significant difference in the results of serum FSH measurement when comparing the study groups (Figure 3). The serum level of testosterone decreased significantly in the nicotine-administered mice when compared to the control group (*p*<0.05). Also, the use of aqueous and methanol extracts of corn silk led to a significant increase in this hormone compared to the nicotine group (*p*<0.05; Figure 4).

### Effect of aqueous and methanol extracts of corn silk on sperm count

**Figure 5:**
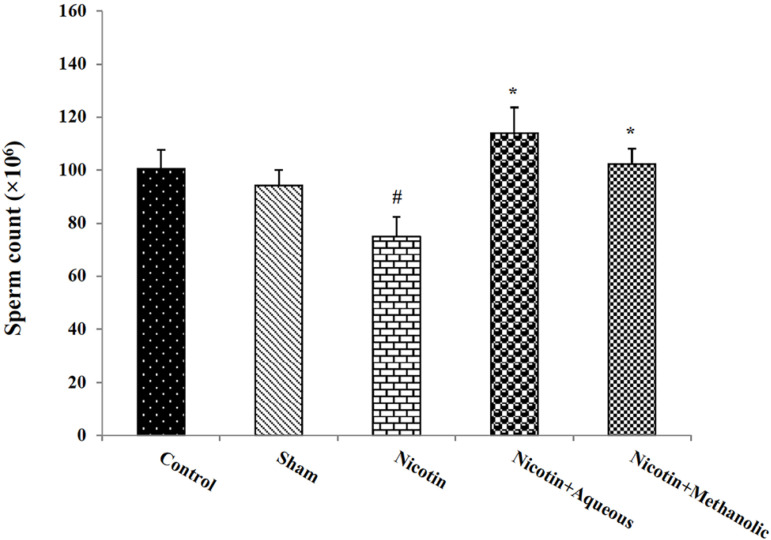
Effects of aqueous and methanol extracts of corn silk on sperm count. The results are Mean±SEM for 6 mice in each group. #: *p*<0.05 compared to the control group; *: *p*<0.05 compared to the nicotine group.

The number of sperm in the nicotine group decreased significantly compared to the control group (*p*<0.05). In addition, this variable showed a significant increase in the nicotine-treated mice with aqueous and methanol extracts of corn silk compared to the nicotine group (*p*<0.05; Figure 5).

### Effect of aqueous and methanol extracts of corn silk on MDA and GSH of testicular tissue

**Figure 6:**
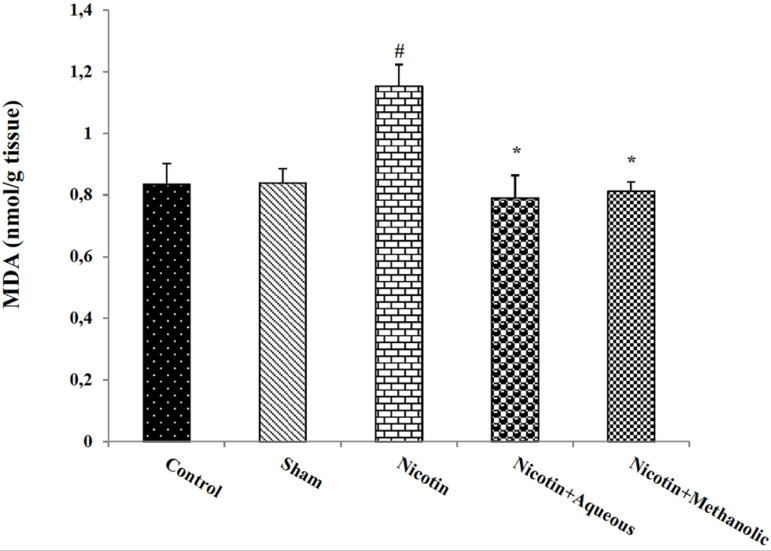
The effect of aqueous and methanolic extracts of corn silk on the amount of MDA in testicular tissue. The results are Mean±SEM for 6 mice in each group. #: *p*<0.05 compared to the control group; *: *p*<0.05 compared to the nicotine group.

**Figure 7:**
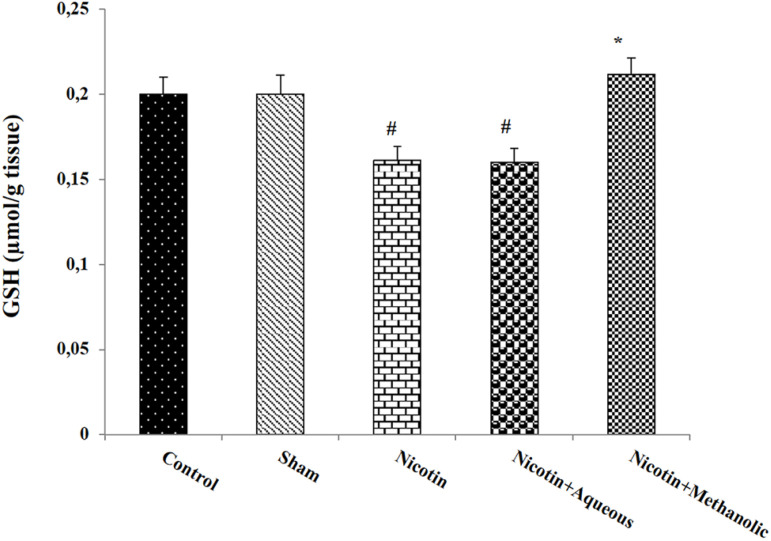
The effect of aqueous and methanolic extracts of corn silk on the amount of GSH in testicular tissue. The results are Mean±SEM for 6 mice in each group. #: *p*<0.05 compared to the control group; *: *p*<0.05 compared to the nicotine group.

The results of the MDA assay in the testicular tissue of the nicotine-receiving group showed a significant increase compared to the control group (*p*<0.05). This variable decreased significantly in the nicotine + aqueous and methanol extracts of corn silk groups compared to the nicotine group (*p*<0.05; Figure 6). Testis GSH levels decreased in the nicotine and nicotine + aqueous extract of corn silk groups compared to the control group (*p*<0.05). Also, this variable showed a significant increase in the nicotine + methanol extract of corn silk compared to the nicotine group (*p*<0.05; Figure 7).

### Effect of aqueous and methanolic extracts of corn silk on testis tissue changes

**Figure 8:**
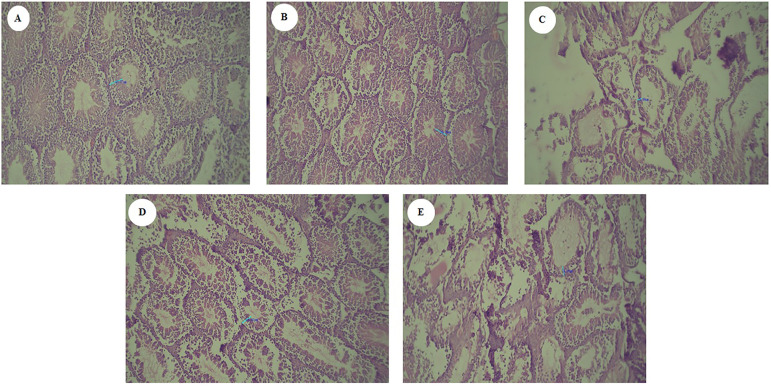
The effect of aqueous and methanol extracts of corn silk on changes in testicular tissue. A: control; B: sham; C: nicotine; D: nicotine + corn silk aqueous extract; E: nicotine + corn silk methanolic extract.

The histomorphometry assessment showed that there was a significant decrease in the thickness of the epithelium of seminiferous tubules in the group receiving nicotine compared to the control group. Microscopically, the number of spermatogenic tubes lost their normal shape and decreased. Also, the amount of interstitial space increased and discontinuity was visible between the tubes and sperm cells. These changes in the testicular tissue of mice receiving nicotine treated with aqueous and methanol extracts of corn silk were improved, and the thickness of the epithelium of the seminiferous tubules increased (Figure 8).

## DISCUSSION

The results of the present study indicated that the use of nicotine after increasing lipid peroxidation and reducing glutathione in the testicular tissue led to increased LH, decreased testosterone, sperm count, and damage to the testicular tissue in mice. On the other hand, the use of aqueous and methanol extracts of corn silk normalized serum testosterone and LH levels, sperm count, testicular MDA, and tissue structure. However, the aqueous extract could not restore testicular GSH to a normal level, but the methanolic extract showed a favorable effect in this regard. Therefore, it can be suggested that the aqueous extract of corn silk probably leads to the improvement of lipid peroxidation in the testicular tissue through other pathways of the antioxidant defense system, which requires more studies in this field.

It has been reported that nicotine prevents the release of FSH and LH hormones from the pituitary gland. Additional research has also shown that nicotine as well as cotinine (a metabolite of nicotine) reduces the concentration of these hormones in rats. More recent studies have shown that nicotine affects spermatogenesis by reducing semen quality, and destruct the normal pituitary hormone-gonadotropin axis action (Hasanudin *et al*., 2012; Mosadegh *et al.,* 2017). In addition, Nesseim *et al*. (2011) showed that nicotine has a doseand time-dependent adverse effect on spermatogenesis. In vivo, nicotine is oxidized to its metabolite cotinine, which has a long half-life, and both nicotine and cotinine negatively affect spermatogenesis, epididymal sperm count, motility, and fertilization potential of sperm. Most research findings have shown that nicotine reduces testosterone levels by inhibiting multiple steps of testosterone biosynthesis in rats and mice. Recently, it has been established that the toxic effects of nicotine consumption occurred due to increased ROS production (Jana *et al*., 2010).

According to the histological results of the present study, the depletion of germ cells by nicotine consumption may be caused by the low concentration of testosterone inside the testis, because the high level of testosterone in the testis is necessary for normal spermatogenesis and also for maintaining the structural morphology and normal physiology of the seminiferous tubule. Therefore, with a decrease in the level of plasma testosterone, the production of mature and normal sperm decreases and leads to a decrease in the sperm number. Testosterone is required for the connection of different generations of germ cells in the seminiferous tubules. Therefore, the low level of this hormone may lead to the separation of germ cells from the epithelium of the seminiferous epithelium and then cause the death of germ cells (Jana *et al*., 2010). Therefore, according to the previous studies and the results of the present study, it can be suggested that the use of nicotine causes a decrease in testosterone and a decrease in sperm count by increasing lipid peroxidation and free radicals in the testicles of animals.

Corn silk is a rich part of corn with high amounts of bioactive components such as flavonoids, polysaccharides, steroids, tannins, alkaloids, proteins and vitamins, which are successfully used as antioxidants in the control of kidney, and prostate diseases (Ebrahimzadeh *et al*., 2008). In the study by Ofoego *et al*., they showed that the ethanolic extract of corn silk has therapeutic potential in reversing paraquat-induced testicular toxicity in rats. Serum testosterone levels, sperm motility, and sperm count were lower in animals that received paraquat alone. These changes occurred as a result of the induction of oxidative stress by paraquat on testicular tissue. The use of ethanol extract from corn silk led to the improvement of the above-mentioned cases by improving the performance of the antioxidant defense system, increasing the activity of anti-acid enzymes, and reducing testicular toxicity (Ofoego *et al*., 2020). Therefore, in agreement with the previous studies, in our study, aqueous and methanolic extracts of corn silk led to improvements in the levels of testosterone and LH hormones, and in the sperm count of the treated mice through reducing lipid peroxidation and improving glutathione.

In a human study, male hypogonadism is characterized by a lack of testosterone. Low testosterone levels may be due to abnormalities of the testes, hypothalamus, or pituitary gland. Testosterone secreted by the testes in response to LH has a negative feedback on anterior pituitary LH secretion. Much of this inhibition is likely due to a direct effect of testosterone on the hypothalamus to reduce gonadotropin-releasing hormone (GnRH) secretion. Therefore, any change in the testicle due to acquired damage, including oxidative stress and induction of toxicity, can lead to a decrease in the normal function of Leydig cells in their secretion of testosterone, and this inhibitory effect is removed from the pituitary gland, which is followed by an increase in the secretion of facial LH hormone. This condition is known as primary male hypogonadism (Hall & Hall, 2020; Kumar *et al*., 2010). Therefore, similar to humans, the results of hormonal tests in the present study indicate that nicotine injection in mice may cause primary hypogonadism in these animals following the decrease of testosterone and increase of LH, and the use of corn silk aqueous and methanol extracts neutralize this change and protect the testicle from inducing such a disorder. Finally, by using the results of the present fundamental-experimental study, it is possible to understand the effects of corn silk extract on the male reproductive system. Furthermore, by conducting clinical research and achieving its non-toxic doses in patients, it can be suggested that the extracts of this plant can be used as an herbal medicine for treating male infertility in people who use nicotine in various ways, including smoking.

## CONCLUSION

In conclusion, the results of the present study showed that the consumption of nicotine led to increased MDA and decreased GSH in the testicles of treated animals, and subsequently, by creating oxidative stress in this organ, it had a reducing effect on the serum testosterone level and sperm count, and increased LH levels. These changes can indicate the induction of primary hypogonadism in small laboratory mice, and we require more studies in this field. On the other hand, the use of aqueous and methanolic extracts of corn silk has removed the oxidative damage caused by nicotine consumption via reducing lipid peroxidation. Also, these extracts have improved the levels of sex hormones and sperm count in animals receiving nicotine. So, according to past studies and the effects of flavonoids in the treatment of male infertility through improving the antioxidant function, it can be suggested that the flavonoids in the corn silk extract have improved the antioxidant defense system of the testis and subsequently the hormonal and reproductive function in male mice. Hence, thin layer or column chromatography (TLC) methods are suggested to separate this compound in future studies.
